# Compliance with epidemic-prone diseases surveillance and response guidelines among health officers at surveillance units in South-West Nigeria

**DOI:** 10.4314/ahs.v18i2.28

**Published:** 2018-06

**Authors:** Magbagbeola David Dairo, Daniel Ola Afolayan, Joshua Odunayo Akinyemi

**Affiliations:** Department of Epidemiology and Medical Statistics, Faculty of Public Health, College of Medicine, University of Ibadan, Ibadan, Oyo State, Nigeria

**Keywords:** Epidemic-prone diseases, case definitions guidelines, surveillance and response

## Abstract

**Introduction:**

Although compliance with surveillance guidelines is crucial to epidemic diseases control, determinants of use of these guidelines in Nigeria are poorly documented. We assess health workers compliance and factors associated with the surveillance and response guidelines for epidemic-prone diseases in South-West Nigeria.

**Methods:**

In a cross-sectional study, 199 disease surveillance and notification officers in Oyo state were interviewed using a questionnaire on knowledge of disease surveillance and performance of surveillance activities. Data were analysed using descriptive statistics, chi-square and multiple logistic regression at P= 0.05.

**Results:**

Most surveillance units submitted disease outbreaks reports (81.48% at the health facility and 100% at the local government level). Timeliness and completeness of weekly reporting were 94% and 95% respectively. a quarter (25.9%) adhered to national case definitions guidelines. About 85.7% did laboratory case confirmation while 2.6% did facility level data analysis. Predictors for six months reporting activity include attending a training on surveillance and reporting (OR=7.92; CI=1.65–37.92), fund adequacy (OR=27.81; CI=7.68–100.60) and knowledge of surveillance dataflow (OR=4.80; CI=1.64–14.10).

**Conclusion:**

In addition to provision of adequate financial and laboratory resources, surveillance activities would benefit from continuous training on surveillance data flow.

## Introduction

The prevention, control and reduction of mortality from epidemics are dependent on an effective disease surveillance system.^1^,^2^ The National Technical Guideline for Integrated Disease Surveillance and Response (IDSR) seeks to ensure the performance of core surveillance activities at all surveillance units (health facilities, local government health departments, states and national epidemiology unit).^3^ The guideline specifies the core activities of infectious disease surveillance and response system to include: detection, confirmation and registration of cases of diseases; disease reporting; data analysis and interpretation; feedback; epidemic preparedness and responses to outbreaks. 3,4 It also specifies that health authorities should provide support for the surveillance and response system such as standard guidelines, reporting forms, training, funding, supervision, logistic resources.^3^

Epidemic-prone diseases (EPD) in Nigeria include cholera, cerebrospinal meningitis, diarrhoea with blood (shigella), measles, yellow fever, viral hemorrhagic fevers (such as lassa fever) and highly pathogenic avian influenza.^3^ These diseases keep occurring and cause the highest case fatality rate in Nigeria.^5^ Nigeria has been faced with repeated out-breaks of these epidemic-prone diseases in recent years.^6^–^10^ Meanwhile, the surveillance of these diseases has been recognized to be weak in Nigeria.^1^,^10^–^14^ This study aims to assess compliance with the surveillance and response guidelines for epidemic-prone diseases in South-West Nigeria.

## Methods

### Study design

A cross sectional descriptive study of the surveillance workers for epidemic prone diseases (EPD) at surveillance units was conducted.

### Study population

The study population was the disease surveillance and notification officers throughout Oyo State. This population includes the state epidemiologist at the state Ministry of Health. At the local government level the population included disease surveillance and notification officers. At the health facility level, the population of disease surveillance officers included the health workers (focal persons) who were assigned the responsibility of reporting on the disease patterns and out-breaks that occurred at the health facility and reporting of out-breaks of diseases within the catchment areas of their health facility.

### Sample size determination

The minimum sample size for the study was derived using the formula for a simple descriptive cross-sectional study as follows:

n=z^2^ pq/d^2^

where

n = calculated sample size,

z = standard normal deviate at 95% confidence interval=1.96,

p = proportion of Disease Surveillance and Notification Officers that regularly report surveillance data to the state epidemiology unit=0.858,1

q = 1 - p, and d = 5% precision of the estimate of the sample size.

After substitution of the relevant figures a sample size of n=188 surveillance officers was obtained. A projected non-response rate of 5% was then included to derive a minimum sample size of 198 surveillance officers.

### Sampling procedure

A four stage sampling was used for selecting the respondents. In the first stage, Oyo state was randomly selected by simple balloting from the list of the six states in South-West Nigeria. The states in South-West Nigeria are Ekiti, Lagos, Ogun, Osun, Ondo and Oyo states.

In the second stage, three local government areas were randomly selected by balloting from the sample frame of local governments in each of the three senatorial districts (North, Central and South) in Oyo state. After the balloting the following local government areas were selected; Kajola, Ogbomosho south, and Surulere Local government areas were chosen from Oyo North Senatorial district; Afijio, Akinyele, and Egbeda Local government areas from Oyo Central Senatorial district while Ibadan North, Ibadan SouthEast and Ibarapa East Local governments areas were selected from Oyo South Senatorial district.

In the third stage, seven wards each were randomly selected by balloting from the list of the health wards in each of the local government areas. In the fourth stage, three health facilities were randomly selected by balloting from each of the wards. Only the consenting health workers who are assigned the duty of reporting diseases occurrence in the health facilities were interviewed. This study interviewed a total of 199 officers at various levels of surveillance activities in the state as follows. Thus the respondents interviewed are drawn from the units of surveillance as follows; the State epidemiology unit (n=1) represented by the State epidemiologist, the local gove rnment health departments (n=9) represented by the disease surveillance and notification officers (DSNO) and at the health facilities (n=189) represented by the focal persons.

### Data collection instrument

Data was obtained by the use of questionnaires, checklists, and records review of the monthly and weekly reporting surveillance data from surveillance units at the Oyo state Ministry of Health and the local government health department.

The questionnaires were derived from the review of the literature on the subject matter and sought information on socio-demographics of the respondents, knowledge on disease surveillance by the officers at the surveillance units. The questionnaire was self administered since all the participants in the study could read and write in English.

Meanwhile, a checklist was used to obtain information on performance of core surveillance activities and support functions. The checklist was used to review the health facility out-patient register, log books and copies of laboratory reports at health facilities, copies of completed IDSR forms, tables or charts showing analysis of EPD, and also to ascertain the availability of standard case definition poster and logistic resources at the surveillance units.

Timelines and completeness of weekly reports in the selected local government areas for the year were obtained from the Epidemiology Unit of the Ministry of Health, Oyo state. Timeliness of reporting was defined as the submission of surveillance reports on or before the stipulated time for submission which is specified by the epidemiology unit of the state. Completeness of reporting was defined as the proportion of expected reports that were received at the end of the cut-off date.^15^

Epidemic preparedness was assessed by determining the presence of an Epidemic Preparedness and Response (EPR) team, Epidemic Management Committee (EMC), written EPR plans, availability of stocks of drugs and material supplies for out-break response at the State epidemiology unit and local government health departments. Data were analysed using descriptive statistics, chi-square and multiple logistic regression at P= 0.05 on SPSS version^20^.

### Ethical approval

Ethical approval for the study was obtained from the Oyo State Ethical Review Committee. The confidentiality of the respondents was assured. The respondents were informed of their freedom of refusal to participate in the study. The study was conducted in accordance with the Helsinki Declaration of 1975, as revised in 2000.

## Results

About half of the surveillance workers were trained on disease surveillance. (Table 1)

**Table 1:** Socio-demographic characteristics of study respondents at epidemiological units

Characteristics		N=199	%
**Age**			
	20–29 years	34	17
	30–39 years	69	34.7
	40–49 years	46	23.1
	50 years and above	50	25.1
**Gender**			
	Males	69	34.7
	Females	130	65.3
**Marital status**			
	Married	159	79.9
	Single/Others	40	20.1
**Occupation (cadre)**			
	Nurses	109	54.8
	Medical Records officers	41	20.6
	Doctors	37	18.6
	Laboratory scientist	2	1.0
	Others*	10	5.0
**Length of service**			
	1–5 years	26	13.1
	6–10 years	55	27.6
	11–20years	55	27.6
	21–30 years	63	31.7
**Type of Surveillance unit**			
	State epidemiology unit	1	0.5
	Local government health department	9	4.5
	Primary health care centre	94	47.3
	Private health facility	91	45.7
	Secondary health care centre	4	2.0
	Tertiary health care centre	0	0.0
**Training on disease surveillance**			
	Trained	98	49.2
	Not trained	101	50.8

* State epidemiologist / DSNO's

Disease reporting was carried out by 154 (81.48%) surveillance units at the health facility level and all surveillance units at the local government health department level. However, in the period of six months preceeding the survey, only about three-quarter regularly submitted monthly surveillance data report.(Table 2) Reporting timeliness for weekly surveillance data was high (94%) while completeness was also high (95%). The State epidemiology unit regularly submitted monthly and weekly surveillance data to the Federal epidemiology unit, provided feedback to all the DSNO's, analysed data for EPD, and epidemic preparedness was good.

**Table 2:** Compliance with epidemic-prone disease surveillance and response guidelines at health facilities and Local government health departments

Guidelines	Health facilities		Local government health departments	
	
	(n=189)	%	(n=9)	%
**Case Identification**				
Utilise Standard case definition	49	25.9	NA *	−
**Case confirmation**				
Utilize laboratory confirmation	162	85.7	NA	−
**Case registration**				
Register cases	180	95.2	NA	−
**Disease Reporting**				
Submit regular monthly report (in the past 6months)	144	76.2	9	100
Have a reporting practice	154	81.4	9	100
**Feedback**				
Provide feedback to health		NI	9	100
facilities submitting report	NI **			
**Analyse EPD data**				
Analyse data within health facility	5	2.6	7	77.8%
**Epidemic preparedness &** **response**				
Presence of EPR team	NA	−	9	100
Presence of EMC	NA	−	0	0
Written EPR plan	NA	−	0	0
Stocks of drugs	NA	−	0	0
Stocks of material supplies	NA	−	9	100

* Not Applicable

** No Information

Less than half of the surveillance workers had received supervisory visit (35.7%) and feedback (45.7%) from higher health authorities. At the health facility level, 25.9% adhered to case definitions guidelines as specified in the national guideline. About 85.7% used laboratory findings for case confirmation, and 2.6% performed analysis of the collected surveillance data at their health facility level before submission to state health department. Meanwhile, less than two-thirds (62.3%) of the surveillance workers reported that funds for surveillance activities were adequate.(Table 3)

**Table 3:** Surveillance support functions available to the surveillance worker at surveillance units

Support functions	Available	Inadequate	Not Available
**Knowledge on pathway** **of dataflow**	110 (55.3%)		89 (44.7%)
**Standard case definition**	62 (31.2%)	-	137 (68.8%)
**Supervision visit**			
(From higher health authorities)	71 (35.7%)	-	128 (64.3%)
**Feedback**			
(From higher health authorities)	91 (45.7%)	-	108 (54.3%)
**Logistic resources available**			
IDSR Form (003)	69 (34.7%)	-	130 (65.3%)
Adequate stationery	171 (85.9%)	25 (12.6%)	3 (1.5%)
Motor vehicle	133 (66.8%)	-	66 (33.2%)
Computer	82 (41.2%)	-	117 (58.8%)
Printer	41 (20.6%)	-	158 (79.4%)
Generator	169 (84.9%)	-	30 (15.1%)
Telephone	195 (98.0%)	-	4 (2.0%)
Calculator	176 (88.4%)	-	23 (11.6%)
**Adequacy of funding**	124 (62.3%)	37 (18.6%)	38 (19.10%)

Predictors for compliance (regular adherence) with monthly reporting guideline for 6 months were training (OR=7.92; CI=1.65–37.92), adequacy of funds (OR=27.81; CI=7.68–100.60), knowledge on pathway of data flow (OR=4.80; CI=1.64–14.10), and 21–30 years of service (OR=6.41; CI=1.36–30.31).(Table 4)

**Table 4:** Predictors for 6 months reporting compliance at surveillance units

Variable	Categories	OR	95% C.I	p-value
Marital status	Others**	Ref		
	Married	1.889	0.361 – 9.990	0.452
Length of service	1–10 years	Ref		
	11–20 years	1.185	0.333 – 4.223	0.793
	21–30 years	6.412	1.357 – 30.309	0.019*
knowledge on pathway	No	Ref		
of dataflow	Yes	4.804	1.636 – 14.104	0.004*
Reporting form (IDSR 003)	Available	Ref		
	Not available	1.211	0.172 – 8.506	0.847
Received Training	No	Ref		
(on disease surveillance)	Yes	7.917	1.653 – 37.919	0.010*
Receive Supervision	Yes	Ref		
(from higher health authorities)	No	1.500	0.361 – 6.226	0.577
Receive Feedback	No	Ref		
(from higher health authorities)	Yes	1.357	0.381 – 4.825	0.637
Motor vehicle	Not available	Ref		
	Available	1.119	0.386 – 3.245	0.836
Stationery	Inadequate	Ref		
	Adequate	5.325	0.952 – 29.777	0.057
Funds	Inadequate	Ref		
	Adequate	27.805	7.683 – 100.6	<0.001*

** single/widowed

* statistically significant

## Discussion

Compliance with the EPD surveillance and response guidelines was higher at the State and local government level than at health facilities. This was similar to what was obtainable in other African countries.^16^,^17^ Compliance with Epidemic Prone Diseases surveillance guidelines on the utilisation of standard case definition was found in only about a quarter of the health facilities; which was lower than the findings in the 2009 report on integrated disease surveillance and response (IDSR) in Nigeria in which only a third of the health facilities had available in the health facility standard case definitions posters for any priority disease.^18^ These differences are perhaps due to differences in methodology of assessing the compliance with the guidelines. In the Kaduna study; the compliance with epidemic prone diseases was assessed based on the presence of a poster on each of the epidemic prone diseases within the health facility. In this current study, compliance to the guidelines was based on reported use of the guidelines by the staff of the health facility. It is expected that presence of a poster showing the guidelines does not necessarily translate to its use by the health officers, hence the lower compliance rate found in this study.

In this study, also almost half of the surveillance units had no standard case definition posters. The findings in our study were similar to what was reported in Tanzania which revealed that provisions of standard case definition guidelines to health facilities were insufficient.^19^ These findings were further corroborated by other studies in Tanzania and previously in Nigeria, which found that majority of the health facilities do not have any case definition poster or booklet for any of the priority diseases.^15^,^20^ Although a high percentage (about four-fifth) of the respondents claimed to resort to laboratory investigation for confirmation of cases of epidemic prone diseases, this finding still indicates a gap in the laboratory support for surveillance at the local level. This finding, however is in contrast to the report of Sahal et al in Khartoum state of Sudan which reported that all health facilities had a functioning laboratory.^16^

Registration of cases in clinic registers in this study was similar to the findings in Uganda which revealed that 92% of health facilities had clinic registers.^21^ Sahal in Sudan had also reported that all health facilities in Sudan had clinic registers.^16^ Furthermore Sow et al also showed that clinical registers were available in more than 95% of all health facilities in Cape Verde^17^. Thus the similarity observed in this study is probably due to the fact that registration and diagnosis are routine practices in all health facilities.

The submission of disease reporting data by the surveillance officers was found to be very high (81.5%) in this study. Lower rates of submission were found in studies in other parts of Nigeria. In the Northern parts of Nigeria, a study in Kaduna state reported that only 57% submitted their surveillance reports to the state ministry of health within 6 moinths of the survey. In Yobe, another Northern state of Nigeria, 70.9% of the surveillance officers submitted their reports to the state office within 6 months of collection.^22^,^23^ These two states are in the Northern part of the country and were known to have shortfalls in employment of medically qualified officers of health. Thus these differences observed could be due to the employment of medical officers of health in all the local government health departments in Oyo State. The medical officers of health were all postgraduate degree holders in Public Health and had the statutory duties to ensure mandatory reporting of notifiable diseases.

However, compliance with regular weekly reporting of EPD over six months period was high (about four-fifths) among the surveillance officers and was similar to the findings of Dairo et al, on weekly reporting in both Ekiti and Osun states.^1^ Both of these states are also South Western Nigeria states, that had also adopted medical officers of health supervision of the process of reporting notifiable diseases in their territories. The high rate of completeness of weekly reporting (95%) found in this study is similar to 92% found among the surveillance officers in health districts of eight selected African countries (Cape Verde, Eritrea, Ethiopia, Lesotho, Gambia, Guinea-Bissau, Malawi and Uganda).^17^

Meanwhile, the timeliness of reporting in this study was found to be very high (94.5%). This is in contrast to the 47% timeliness of reporting found among surveillance officers in the health districts in Tanzania and higher than the 85% reported among the surveillance officers in health districts of other eight selected African countries (Cape Verde, Eritrea, Ethiopia, Lesotho, Gambia, Guinea-Bissau, Malawi and Uganda).^15^,^17^ The reported differences could be attributed to the financial support to the surveillance officers in Oyo State by the World Health organization to ensure zero reporting strategy. Thus all disease surveillance officers and focal persons are motivated to comply. Thus the timeliness of reporting in Oyo State is commendable as it exceeds the WHO/CDC target of 80% of all facilities.

Only five (2.6%) health facilities analysed surveillance data for any of the epidemic-prone diseases and 46 (24.3%) analysed for at least only malaria. This percentage of surveillance officers engaged in local analysis of data in their facilities is higher than the findings in Kaduna state which reported that only 19% of them did any form of data analysis in their health facilities.^22^ This proportion is also higher than the 10% reported in Uganda but lower than the 32% reported by Mghamba in Tanzania.^19^,^21^ In addition Gueye et al in Tanzania also reported that 33% of health workers in the facilities surveyed did data analysis for priority diseases while 28% did for malaria.^15^ The lack of data analysis at the health facility level could be due to the fact that health workers consider the data collected as just required for forwarding to higher levels rather than for local use. At the local levels, health workers should be able to make tables of frequencies of diseases occurrence and draw graphs of trend of their occurrence.

Although the IDSR criteria on epidemic preparedness and response were met by the State epidemiology unit in this study, epidemic preparedness among the Local governments area health facilities was completely absent. This observation is similar to the finding reported in Sabon Gari Local government area of Kaduna state where epidemic preparedness was poor as less than half of the epidemic preparedness and response criteria were met.^24^ In our study there were no stocks of drugs and materials such as specimen bottles, needles and syringes for outbreak response. This is despite the indication that the local government health departments had standby emergency response team. The lack of emergency preparedness despite availability of emergency response team indicates a disconnection that will delay or hinder the implementation of any emergency response plan.

This study revealed that training of surveillance workers was a predictor for compliance with EPD monthly reporting guideline over six months. Participation in training for surveillance was reported by almost half of the respondents at the health facility level and all the disease surveillance and notification officers at the local government levels. . The salutary effect of training on performance of surveillance functions had been reported in a study by Bawa and Olumide in Yobe state that showed that training positively impacts the disease notification habits of health personnel.^25^

Adequacy of funds, which was found to be a predictor for compliance with EPD monthly reporting guideline is in agreement with the report of a study in Osun and Ekiti state which showed that adequate funding was a predictor for the reporting of out-breaks.^1^

The observation in this study that knowledge of the surveillance dataflow pathway among surveillance workers is a predictor for compliance with the EPD reporting guideline corroborates the findings in Ekiti and Osun state in which it was reported that the knowledge of the pathway of disease notification directs surveillance workers and indirectly predicts the level of awareness of their duty.^1^ This finding also agrees with another study by Sow et al that reports that district health personnel knowledge about the national priority diseases improves reporting completeness and timeliness.^17^

The 21–30 years of service as a predictor for compliance with EPD reporting guideline may probably be due to the fact that these workers might have been exposed to training on disease surveillance during their long length of service. Furthermore increased years of service would enable understanding of roles and responsibilities and development of necessary skills required for the performance of statutory duties. These officers would also have been given leadership and supervisory roles that compels the performance of their duties in a satisfactory manner.

## Conclusion

Compliance with the core surveillance guidelines is good at the state level. However, it is defective at the local government health departments (with respect to poor epidemic preparedness) and at the health facilities (with respect to the utilisation of standard case definitions and health facility based analysis of EPD surveillance data). Furthermore this study reveals the inadequacy of laboratory support for surveillance activities at the local levels. It is recommended that local and State government ensure the provision of continuous training and resources to surveillance workers so as to achieve effective disease control. The strengthening of laboratory support for disease surveillance at the local health facility level has also become imperative.
